# Superposition principle applies to human walking with two simultaneous interventions

**DOI:** 10.1038/s41598-021-86840-9

**Published:** 2021-04-02

**Authors:** Fatemeh Rasouli, Seok Hun Kim, Kyle B. Reed

**Affiliations:** 1 Department of Mechanical Engineering, University of South Florida, Tampa, 33620 USA; 2 School of Physical Therapy and Rehabilitation Sciences, University of South Florida, Tampa, 33620 USA

**Keywords:** Biomedical engineering, Rehabilitation, Scientific data, Musculoskeletal system

## Abstract

Gait rehabilitation therapies provide adjusted sensory inputs to modify and retrain walking patterns in an impaired gait. Asymmetric walking is a common gait abnormality, especially among stroke survivors. Physical therapy interventions using adaptation techniques (such as treadmill training, auditory stimulation, visual biofeedback, etc.) train gait toward symmetry. However, a single rehabilitation therapy comes up short of affecting all aspects of gait performance. Multiple-rehabilitation therapy applies simultaneous stimuli to affect a wider range of gait parameters and create flexible training regiments. Understanding gait responses to individual and jointly applied stimuli is important for developing improved and efficient therapies. In this study, 16 healthy subjects participated in a four-session experiment to study gait kinetics and spatiotemporal outcomes under training. Each session consisted of two stimuli, treadmill training and auditory stimulation, with symmetric or asymmetric ratios between legs. The study hypothesizes a linear model for gait response patterns. We found that the superposition principle largely applies to the gait response under two simultaneous stimuli. The linear models developed in this study fit the actual data from experiments with the r-squared values of 0.95 or more.

## Introduction

Human walking is a complex yet flexible mechanism. It involves hundreds of muscles and bones controlled by elaborate pathways and interconnections of the neuromusculoskeletal system. Damage to this system can significantly decrease functional performance and quality of life^1,2^. Stroke is the third largest cause of disability worldwide^1^, with 80% of the survivors suffering from walking dysfunction^2^. Hemiparesis (hemiplegia), defined as muscle weakness (paralysis) on one side of the body, is a common condition post-stroke^3^. Hemiparesis (hemiplegia) causes asymmetric walking that considerably decreases gait functionality and daily task performance^4,5^. Addressing these problems requires a better understanding of the interconnections between neurons, sensory feedback, and muscles. Identifying how gait reacts to multiple sensory inputs and the behavior of the musculoskeletal system under stimulus is critical for developing appropriate rehabilitation therapies.

Motor adaptation and motor learning skills are the basis for rehabilitation of human motor behavior^6^. Motor adaptation is the process of modifying a well established gait pattern and has a transient nature^5^. The essential goal of motor adaptation is to minimize the error between the brain’s predicted outcome and the actual observed outcome while considering the cost of body movement^5^. Motor adaptation is a short-term effect and is de-adapted when training is removed (post-adaptation). Motor learning is the long-term learning of a new or relearnt pattern that can be easily switched to without requiring training beforehand^7^. While motor adaptation and motor learning interactions have not been fully understood yet, it is highly suggested that repeated motor adaptation and de-adaptation processes over time (days, weeks, or months) can lead to motor learning^5,6,8^; meaning that a temporary walking pattern can become permanent through continuous training. For example, patients with unilateral cerebral stroke have strongly demonstrated short-term motor adaptation capabilities^9^, which can then turn to long-term motor learning through repeated training^8^.

Gait rehabilitation therapies post-stroke can be divided into two main categories: neurophysiology and motor learning approaches^7^. The neurophysiological approach uses a developed technique (such as Bobath^10^) to reconfigure the incorrect gait pattern, and the patients take a passive role in the process. Motor learning techniques, on the other hand, require active participation from patients where they get training (with the help of a physical therapist) to improve their gait performance gradually. These training techniques incorporate different setups, modalities, and sensory inputs to entrain gait using approaches such as strength training^11,12^, treadmill walking^13–16^, rhythmic auditory stimulation (RAS)^17–19^, robot-assisted training^20,21^, and visual biofeedback^22^. However, current rehabilitation therapies are unable to control and retrain all the gait parameters at the same time and in most cases their effects do not last long-term^6,9^. One major reason for ineffective therapies is patients are unable to transfer trained patterns from a rehabilitation facility to their home and everyday life^23^. Other reasons include techniques that lack consideration for individual strengths and weaknesses of the survivors^24^ or therapies that are not entraining pathologic gait for normal walking^25^.

A common training approach for symmetric gait is treadmill walking because it enforces adjusted speed to the user. Inadequate walking speed is a major contributing factor for inefficient gait among stroke survivors^26^. When matching speed on a treadmill, the spatiotemporal differences between post-stroke and healthy matched subjects were reduced^3,4,27,28^. While tied-belt treadmill walking (same speed on both sides) is able to improve walking speed (and increase efficiency as a result), it is not effective in improving gait symmetry long-term. Exaggerating asymmetry of an impaired gait (error augmentation) using a split-belt training (SBT) has shown a better post-training aftereffect^9,23^. This aftereffect can be linked to the compensation mechanism in gait that is applied and stored through proprioceptors (sensory receptors within the muscles) during training^29,30^. For instance, imagine you are driving a car with misaligned wheels pulling the car slightly toward the left. You need to constantly adjust the steering wheel to compensate for the tilted wheels to stay in a straight line. After a while, if you drive a new car, you will notice errors in steering the wheels toward the right when trying to go straight even though your new car is perfectly aligned. This is the effect of error augmentation training, and it is used for augmenting the asymmetry in stroke survivors with the goal of achieving reversed aftereffect (symmetric gait) post-training. While using an SBT has improved gait speed and interleg parameters such as step length^9,23^, it does not show any aftereffect on intraleg parameters such as stride duration^9^. It is possible that the lack of sensory feedback during training may account for the limited effects. In addition, accessing an SBT is not easy and most patients require visits to rehabilitation facilities for training. Another major challenge of this type of training is transferring the adapted patterns of SBT to overground walking and into the everyday home environment^8^.

Rhythmic Auditory Stimulation (RAS) is another common training approach for gait symmetry that can affect a different range of gait parameters with a different impact than SBT. There is a strong connection between auditory feedback and the motor control system throughout the central nervous system (CNS)^17^. When a subject is walking, RAS influences them by providing musical rhythms to cue motor function. Research has tested the effect of RAS with various features, ranging from isochronic^31,32^ to biologically varied^25^ and from metronomes^33^ to music-based beats^34^. Previous research has used RAS in which the cues to the left and right are the same duration. We ran a preliminary study to evaluate the effectiveness of Asymmetric Rhythmic Auditory Stimulation (ARAS) in which the cue duration differs between the left and right legs^35^. The adaptation of each leg has been shown to be independent of the other side^36^. Therefore, we hypothesized that *ARAS is able to train each leg to their cue duration independently*. Auditory stimulation is more accessible than SBT and can be easily used in any environment (like home) through any sound playing device. However, the effectiveness of auditory stimulation varies among patients depending on their rhythmic skills^37^. While some patients have shown significant positive results, others show no or negative results^17,37^.

Rehabilitation therapies using a single intervention (such as auditory stimulation or treadmill training alone) are not capable of creating widespread gait changes that are effective for various patients who demonstrate different aspects of asymmetric gait. The combination of rehabilitation therapies has shown better results than a single rehabilitation therapy^7,26^, giving more ability to control parameter changes and the gait outcome performance as a result. Multiple-rehabilitation therapy includes two or more interventions applied simultaneously with the purpose of enhancing performance by engaging more sensory feedback and increasing the control over adjusting gait parameters. Single rehabilitation therapy can train gait to improve some parameters while making no change or worsening others. A multi-modality approach can increase the outcomes of clinical training and focus on the individual’s needs and capabilities^22,33,38–42^. Combinations of tied-belt treadmill training and symmetric RAS have been shown to enhance gait performance more than symmetric RAS overground^33,40,42^.

**Figure 1:**
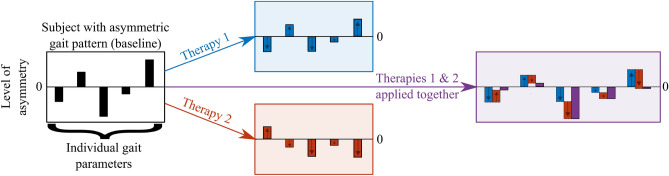
Predicted model of gait behavior under external stimuli. Black rectangle indicates different gait parameters level of asymmetry. Blue and red shaded backgrounds indicate effects from two rehabilitation therapies applied separately. Purple rectangle on the right is the hypothetical behavior of gait under multiple-rehabilitation therapy with therapies 1 and 2 applied simultaneously. Arrows indicate direction of asymmetric effect.

The mechanism of gait response from concurrent external stimuli is not completely understood yet. This study focuses on the interworking of gait performance under two therapy interventions applied simultaneously: treadmill training and rhythmic stimulation. We hypothesize that the superposition principle^43^ applies to gait response; *the net gait response caused by two or more stimuli (rehabilitation interventions) is the sum of the gait responses that would have been caused by each stimulus individually*. Figure 1 demonstrates the predicted behavior of the neuromusculoskeletal system under this hypothesis. Each parameter gets affected differently under different interventions (single rehabilitation therapy). However, if multiple-rehabilitation therapy (combining two interventions) is applied, we predict that gait combines the response with a linear model.

## Methods

### Rationale

To investigate the linearity of the gait response, we constructed our experiment to evaluate two properties of linear systems: (1) additivity examines if the gait asymmetry under multiple-rehabilitation therapy is the sum of the gait asymmetries under single therapies, and (2) homogeneity evaluates if the proportion of asymmetries applied in each therapy are transferred. Evidence from existing research has given glimpses into the related walking mechanisms. We know spatial and temporal gait parameters are accessed through distinct neural pathways^15^ and right and left legs as well as forward and backward walking are controlled through independent networks^36^. However, previous research has not yet studied (as of January 2021) the relationship between how gait responds under single and multiple-rehabilitation therapy.

We propose a combination of SBT and ARAS. Each method creates a type of asymmetry in the gait parameters. We hypothesize that each of these therapies will affect the resulting gait independently of the other. We should also be aware of individual differences between each subject and provide personalized models for the multiple-rehabilitation therapy that takes these differences into account to allow for future customization.

### Experimental design

**Figure 2:**
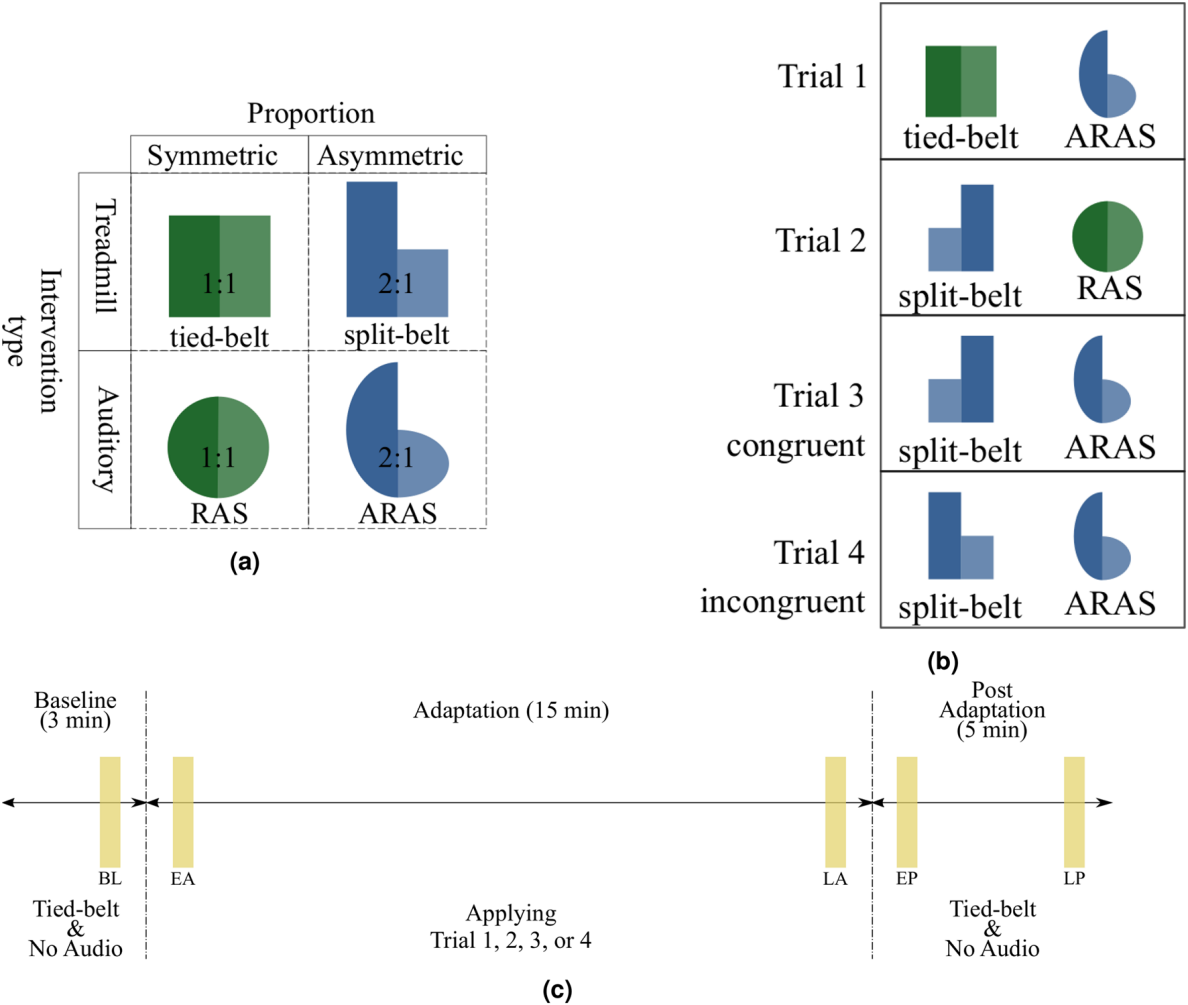
Experimental design: (**a**) Types of therapeutic interventions where height of each shape represents relative magnitude of intervention. (**b**) Therapy interventions that were applied in each trial. Trial 3 and 4 both used two asymmetric 2:1 (blue) therapy interventions. In order to test the effect of the error augmentation mechanism, SBT and ARAS were once applied congruently (trial 3) and once incongruently (trial 4). (**c**) Timeline: duration of each section in the experiments. Yellow rectangular shapes show the approximate location of time windows used for analysis. Each window includes 25 left and 25 right steps. The time windows are located after the first 10 steps or before the last 10 steps of each phase. *BL* baseline, *EA* early adaptation, *LA* late adaptation, *EP* early post-adaptation, *LP* late post-adaptation.

We conducted a prospective cohort study to test our hypothesis. Each subject completed four different trials. Trials were at least 24 hours apart to make sure the residual effects from the previous trial had washed out. We applied multiple-rehabilitation therapy by combining treadmill training and auditory stimulation (Fig. 2a). Each trial incorporated one out of the four combinations depicted in Fig. 2b. We used two interventions with two different proportions (1:1 and 2:1). For both asymmetric interventions, we used a 2 to 1 ratio between left and right to be consistent with previous research^9,23^. In trials 1 and 2, only one of the therapies had an asymmetric 2:1 pattern (blue shapes) while the other stayed symmetric (green shapes). In trials 3 and 4, both therapies were applied with an asymmetric ratio of 2:1. In trial 3, the asymmetric ratios were matched congruently where the faster belt was on the same side of the longer cue. In trial 4, the asymmetric ratios were matched incongruently, where the faster belt was on the same side of the shorter cue. Figure 2b shows the experimental design of each trial. We refrained from doing a trial with two symmetric (green) therapies, as this has been explored in previous research^33,40^ and would not provide additional information for modeling the gait response.

### Participants

Twenty healthy subjects were recruited initially in the study. Two subjects did not complete all the visits/trials, and their data were removed for consistency. One subject was also removed due to errors in the recording from the force plates. Another subject was not included because there were problems with marker drop during the experiments more than one time, and we refrained from redoing the experiments more than once due to the learning effect. In total, 16 healthy subjects (5 females; mean weight = 70.5 kg, SD = 13.0; mean height = 170.9 cm, SD = 9.6) with no prior history of gait impairment and no gait injury in the past 12 months formed the final group. The experimental protocol and the consent form were approved by the University of South Florida’s Institutional Review Board. Written informed consent was obtained from all the subjects prior to participation. All research was performed in accordance with the relevant guidelines and regulations. We divided the subjects into two equal groups of eight. Each completed all four trials (Fig. 2b) in random order. The side of the faster tread was switched in trial 2: left belt was faster for group A and the right belt was faster for group B.

### Definitions, data collection, and procedure

In this study, we use the term ‘tied-belt’ when the treadmill belts have the same speed and ‘split-belt’ when each belt has a different speed. Percent asymmetry is defined as ‘left–right’ divided by the sum of left and right. We chose this definition over ‘slow–fast’ because the side of asymmetry in the trials is important for the purpose of this study. We calculated 5 time windows to compare the results of each trial. Each time window is the average of asymmetry between 25 left steps and 25 right steps (50 consecutive steps). Each subject took a few steps to adjust their walking after the start or stop of the training. Therefore, the time windows are located just after the first 10 steps or before the last 10 steps of each phase to make sure gait reaches stability (we first averaged the steps within each subject, then averaged the overall group).

All experiments and data recording were conducted using the Computer Assisted Rehabilitation ENvironment (CAREN) system. CAREN system contains an immersive virtual reality environment, a 3D motion capture system, 6-degree-of-freedom motion base, instrumented dual-belt treadmill, and integrated force-plates. We used reflective markers on the lower body joints including metatarsal, heel, and lateral malleolus of the ankle to keep track of lower limb movements. Force-plates under the treadmill captured ground reaction forces (GRF). Sound cues were played through surround audio speakers mounted on the ceiling of the safety cage. Each cue was the recorded standard dictionary (American English) pronunciation of the word ’right’ or ’left’. Subjects were instructed to adjust each foot landing with their respective cue. Data were recorded at 100 Hz frequency. Prior to the first trial, subjects practiced on the tied-belt treadmill until they felt comfortable (approximately 3 minutes). During this time, they were asked to choose their comfortable speed while the operator changed the speed of the treadmill by increments of 0.1 m/s. Then the step time of the tied-belt walking was calculated by averaging 10 consecutive steps on the treadmill with the set comfortable speed. The fast belt was calculated by 4/3 of the comfortable speed, and the slow belt was half of the fast belt (2/3 of the comfortable speed) to keep the same average speed with a 2:1 ratio. The same process was implemented for the duration of asymmetric cues in ARAS. The cues are spaced isochronously based on the step time. For instance, if the average step time of a subject is 750 ms, a 2:1 ratio of ARAS has a 1000 ms cue on one side and a 500 ms cue on the other. Each trial took 23 minutes to complete (Fig. 2c) and had three phases: 3 minutes of baseline (tied-belt and no sound), 15 minutes of adaptation using one of the trials 1 through 4 combinations, and 5 minutes of post adaptation (tied-belt and no sound).

### Data analysis

This study was a within-subject design. Multiple linear regression analysis was conducted to test the hypothesis using two explanatory variables (trials 1 and 2) to estimate the outcome of two dependent variables (trials 3 and 4). Statistical analysis was performed using IBM SPSS Statistics 26. The regression analysis reported the r-squared values, the significance levels using *p* value (0.05), and the collinearity diagnostics of the linear model. Each coefficient of the model was calculated based on 30 data points (2 trials $\times$ 3 gait parameters $\times$ 5 time windows).

## Results

### Gait parameters

**Figure 3:**
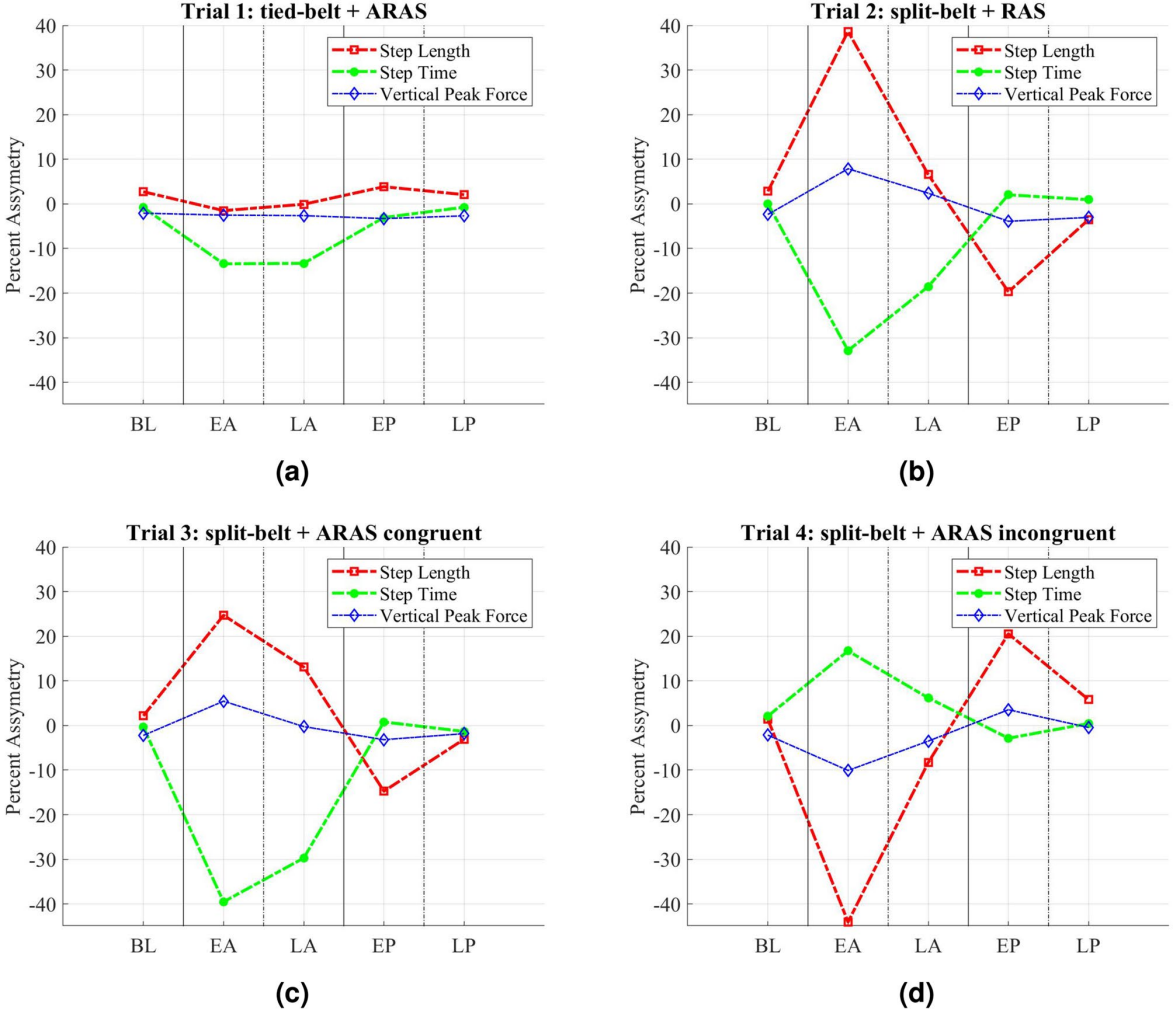
Percent asymmetry average of step length, step time, and peak vertical force during five time-windows (*BL* baseline, *EA* early adaptation, *LA* late adaptation, *EP* early post-adaptation, *LP* late post-adaptation) for group A. Solid vertical lines indicate the start and stop of each phase. (**a**) Trial 1: tied-belt + ARAS, (**b**) Trial 2: SBT + RAS, (**c**) Trial 3: SBT + ARAS congruently, (**d**) Trial 4: SBT + ARAS incongruently. In trials 1 and 2 (top row) only one intervention was applied asymmetrically while both interventions were asymmetric in trials 3 and 4 (bottom row).

Subjects had an average comfortable speed of 0.91 m/s (SD = 0.21) with average step time of 0.63 s (SD = 0.093). Spatiotemporal parameters, as well as GRF, were calculated. Figure 3 indicates the average of percent asymmetry for step length, step time, and peak vertical force for group A during the three phases of the experiments (baseline, adaptation, post-adaptation). Figure 3a shows trial 1 (tied-belt+ARAS) has a larger effect on step time compared to step length asymmetry. Auditory sensory feedback has been shown to influence largely the temporal parameters in the gait^44^. Trial 2 (split-belt+RAS) in Fig. 3b affects both step length and step time (step length slightly more than step time). This result is also in accordance with previous findings that SBT engages interlimb parameters^23^. Trials 3 (congruent) and 4 (incongruent) use both SBT and ARAS effects at the same time. Figure 3c with the congruent combination is indicating a sum effect of trials 1 (tied-belt+ARAS) and 2 (split-belt+RAS), while Fig. 3d with the incongruent combination is indicating the difference of trial 1 (tied-belt+ARAS) minus trial 2 (split-belt+RAS). In all SBT trials, there is an opposite change in asymmetry during early post-adaptation compared to early adaptation, which indicates the neural system has temporarily stored the applied asymmetry^6^. Group B confirms the similar behavior of combined effect and storage of adaptation through overcorrection mechanism. Supplementary Figure 1 indicates the results. The fast and slow belt sides were switched in trial 2 (split-belt+RAS) for group B. Therefore, the result shows a reversed direction for trial 2 (split-belt+RAS).

### Linear model

The superposition hypothesis proposes that the resultant change in gait under two stimuli is the linear combination of changes under each stimulus applied individually. To test the applicability of this hypothesis for gait response under asymmetric interventions, we developed a linear model for the three measurements (step length, step time, and peak vertical force) indicated in Fig. 3. Two asymmetric stimuli, SBT and ARAS, were applied both individually and combined. Trial 1 and 2 included only one asymmetric stimulus, while trials 3 and 4 applied both stimuli asymmetrically. We fitted a model that estimates the gait response for the two later trials based on the first two trials’ gait responses. Equation 1 shows the linear model equations:

1 $$\begin{aligned} \begin{aligned} Trial 3_{estimate}&= C_1*Trial 1+C_2*Trial 2\\ Trial 4_{estimate}&= C_1*Trial 1-C_2*Trial 2 \end{aligned} \end{aligned}$$

**Figure 4:**
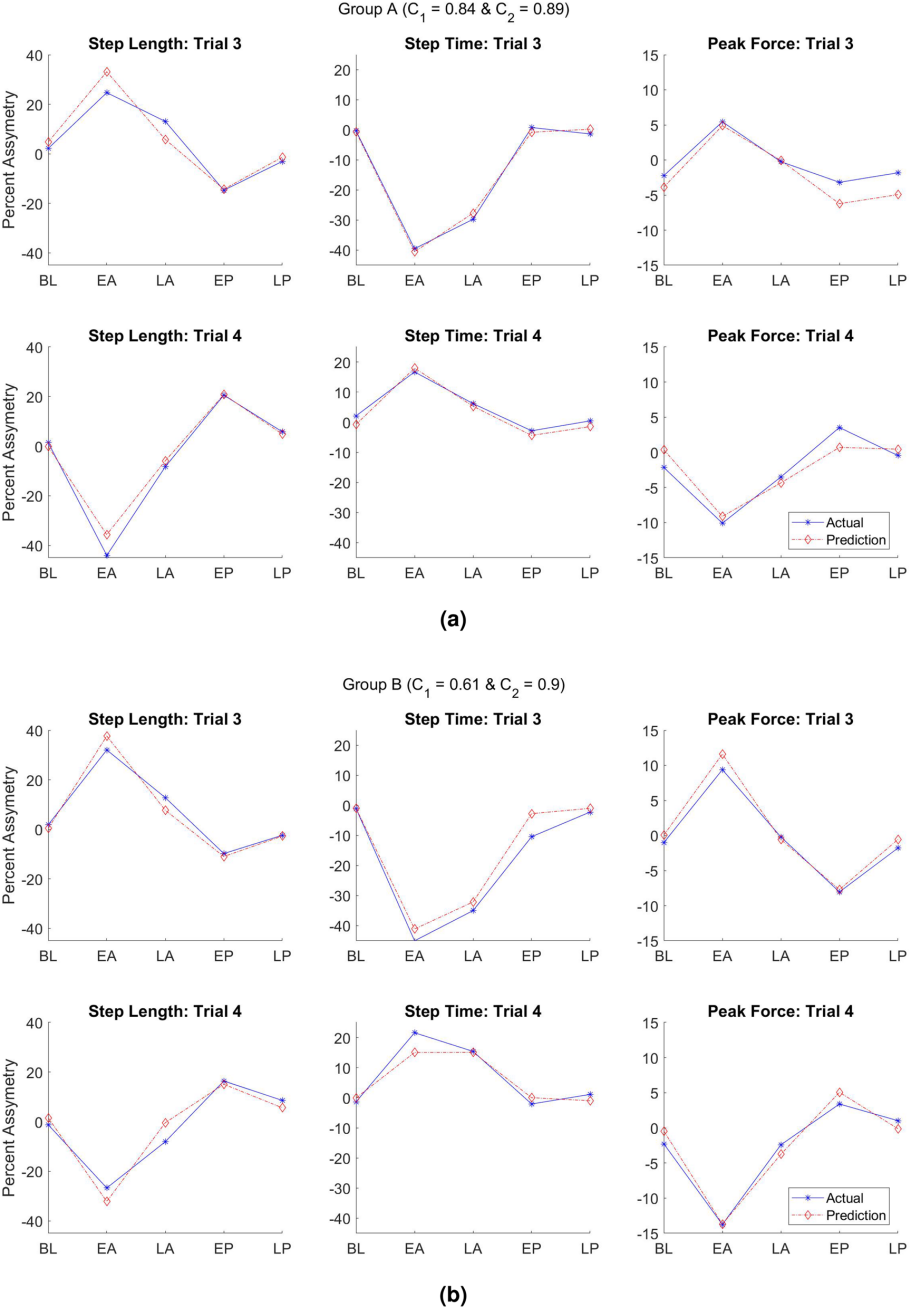
Linear model predicting the asymmetric performance of step length, step time, and peak vertical force compared to real data during trial 3 and 4. The predictions are the dashed red lines, and the actual results from experiments are solid blue lines. (**a**) Group A actual data and model with the coefficients C1 = 0.84 and C2 = 0.89, (**b**) Group B actual data and model with the coefficients C1 = 0.61 and C2 = 0.90. The top row in each group represents the linear model and real data for the congruent combination (trial 3), and the bottom row represents the linear model and real data for the incongruent combination (trial 4).

$C_1$ and $C_2$ are constant coefficients of the model and stay the same in both equations because the interventions were applied with the same ratio in trial 3 and 4. We only changed the direction of applied asymmetry between trial 3 and 4 to create the congruent and incongruent effect. The negative sign in trial 4 estimation indicates this difference. We calculated the two coefficients by minimizing the root mean square error of the models at the same time. Trial 3 is the congruent combination because both directions of asymmetry in ARAS and SBT guide the gait toward the same asymmetric side. Trial 4 is the incongruent combination since the asymmetric direction of SBT and ARAS guide the gait toward opposite sides of asymmetry. We compared the result of the fitted model to the actual data (Fig. 4a,b) for both groups A and B. This linear model estimates the behavior of three gait parameters in spatial, temporal, and kinetic areas over the three phases of the experiments for two combinations of asymmetric interventions.

[$C_1$, $C_2$] values were [0.84, 0.89] for group A and [0.61, 0.90] for group B. The overall r-squared values for group A and B models were 0.96 and 0.95, respectively. The r-squared values indicate the linear model explains more than 95% of variation within the data. The statistical test also calculated the *p* values of variables in the regression model. The p-values of both independent variables (trial 1 and trial 2) were 8.4e-20 in group A and 2.6e-19 in group B, indicating very strong evidence in favor of the hypothesis. The results show that combined asymmetric changes under trial 1 and 2 are significantly associated with changes in the response of trial 3 and 4. No collinearity was found among trials 1 and 2, having values of the variance inflation factor of 1 for all coefficients. While $C_2$ values in both groups were close (0.89 and 0.90), the $C_1$ value for group B was 0.61, indicating less effect of ARAS in group B compared to group A. After close examination, two subjects in group B showed close to zero or negative gait response under trial 1 (tied-belt+ARAS). Running the model for group B without the two outlier subjects gives values closer to group A: $C_1 = 0.77$ and $C_2 = 1.00$. Supplementary Figure 2 also indicates the scatter plots of the multiple linear regression model compared to real data.

### Personalized coefficients

**Figure 5:**
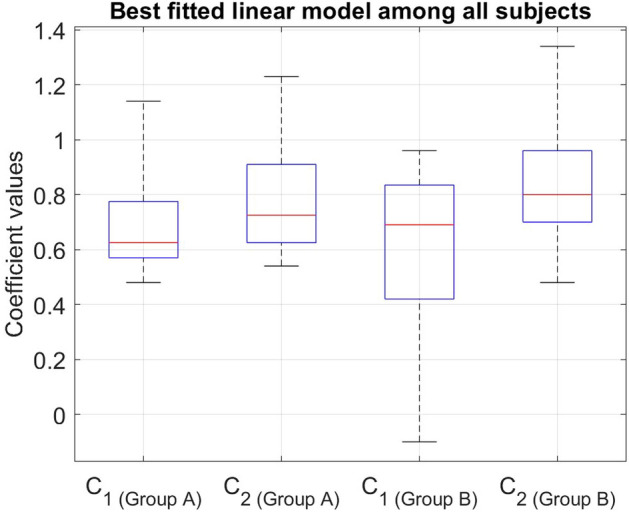
Whisker plot of coefficients for individual linear models.

Our linear model of the gait response applied the superposition principle to asymmetric gait behavior under two simultaneous stimuli for the averaged results of all subjects. While the model presents a great fit for the averaged data supporting the hypothesis, it does not provide the optimal model for each individual. We also calculated personalized coefficients for each subject by minimizing the root mean square error of the linear model (Eq. 1) for each person. Figure 5 indicates the whisker plot of the coefficient values for the optimal personalized linear models. The exact values for all subjects have been reported in Supplementary Table 1. Two subjects in group B had close to zero or negative $C_1$ values for their best fit models (subjects 13 and 15 in Supplementary Table 1). Training based on the auditory sensory feedback have shown none or even negative responses from participants depending on their rhythmic synchronization capabilities^37^. As a result, the first quartile of $C_1$ in group B was stretched to near zero.

## Discussion

Reciprocal movements such as walking contain three stages: initiation, cyclic pattern, and termination of the movement. CNS involvement has been suggested to vary between these stages^45^. While initiation and termination are regulated at the supraspinal level, cyclic patterns are generated at the spinal level by central pattern generators (CPGs) and motor neurons^45–47^. Therefore, the involvement of the supraspinal level in pattern generation is minimal and indirect. While there is strong direct evidence for the existence of CPGs in other vertebrates such as cats^47^, there is indirect yet strong evidence supporting the existence of CPGs in humans^45–47^. Moreover, research has shown that the brain does not control every detail of every movement^26^. Instead, it only controls the endpoint, in this case, the final placement of the foot. It combines groups of muscles into modules for controlling tasks such as balance or walking^26^. Modules simplify control of the coordination of movement by the nervous system and create flexibility within the muscles, sensory feedback, and neurons^48^. In patients suffering from stroke, damage to the brain causes these modules to integrate together in order to make them easier to control^26^. Therefore, the default walking module changes. The default walking module is the preferred walking style when no intervention is applied, whether a person has a healthy or an impaired gait. Now we know that due to the interconnections of the neuromusculoskeletal system, damage to the brain does not mean that the ability to walk normally and create new modules is completely lost^6^. Cerebellar damage limits gait adaptation^49^, but the capability for rehabilitation and gait retraining is retained after damage to the cerebral cortex^5^. Therefore, stroke survivors with cerebral damage are capable of (re)learning symmetric walking patterns. (Re)training the muscles and restoring the pathways of neural control of movement through rehabilitation therapy have shown promising results^8^. However, single rehabilitation therapy comes up short of complete gait restoration.

The literature shows that no single method is most effective for gait retraining^7^. Rather, multimodal approaches are likely to provide better outcomes^26^, and studies have demonstrated the benefits of combined therapies^39–42,50^. The benefits of multimodal approaches could likely stem from the capability of the neural system to independently control different aspects of gait. For example, Kozlowska et al.^15^ found that different neural circuits are responsible for spatial and temporal locomotion control. Choi and Bastian^36^ found that walking adaptations are stored independently for each leg. While most of the multiple-rehabilitation therapies result in better overall performance in participants, there needs to be a framework relating how adaptations merge together in the neuromusculoskeletal system.

We applied two interventions with 1:1 or 2:1 ratios at the same time in four different trials, measuring gait response each time. By combining two rehabilitation therapies, we hypothesized the superposition principle applies to the gait response under combined treadmill training and rhythmic stimulation. Using the ARAS technique, an innovative method developed for the first time; we were able to match 2:1 asymmetry in split-belt with the same asymmetry in sound ratios. Auditory stimulation (RAS or ARAS) has a feedforward mechanism where gait parameters adjust in anticipation of the upcoming cue. In this rehabilitation therapy, the ability to minimize the error between the predicted cue and the actual cue plays an essential role. Treadmill training (split-belt or tied-belt) has a feedback mechanism where the subject reacts to the change of speed or asymmetry, activating interleg control^15^, which adjusts the gait parameters to bring the pattern back to the default walking pattern. While the treadmill trains gait by engaging muscles and proprioceptors into a different walking pattern, auditory stimulation requires conscious attention to auditory sensory feedback.

Modeling gait response can lead to a better understanding of the neuromusculoskeletal system performance under multiple stimuli. A gait response model can also help with developing personalized multiple-rehabilitation therapies that encompass multitudinous aspects of individual capabilities. In this study, we proposed a linear model for the gait response under simultaneous stimuli (interventions) and calculated the percentage of contribution of each stimulus to the final gait asymmetry in each subject. The coefficient values indicate the relative effectiveness of each intervention in the combined trials. A value of one for $C_1$ or $C_2$ indicates the full effect is realized. A coefficient between zero and one would indicate the effect of the intervention was partially transferred. $C_1$ and $C_2$ values for both groups are higher than 0.6 and less than 1, which indicates that less than 40% of the therapeutic effects of each intervention is lost when the therapies applied simultaneously. Three out of the four coefficients are higher than 0.84, meaning the loss of therapeutic effects of their corresponding interventions are 16% or less. For example, $C_1 = 0.85$ and $C_2 = 0.91$ for group A means that 85% of the ARAS effect and 91% of the SBT effect were demonstrated across step time, step length, and peak vertical force during combinations of ARAS and SBT. The high r-squared values and the level of significance confirmed that the suggested model estimates the gait response under two simultaneous stimuli with high accuracy.

While this model shows the average response of all subjects, it might not represent the best estimate for each subject individually. Rhythmic capability, muscle strength, physique, and other personal aspects can affect the gait response of individuals under asymmetric interventions. Level of impairment also change the effectiveness of different rehabilitation therapies between individuals. For this reason, it is important to look at the variability of the model among individuals. It is vital for the improvement of personalized therapies that individual characteristics, especially individual gait response to various inputs from different therapies, to be taken into consideration when prescribing a multiple-rehabilitation therapy.

Calculating personalized coefficients from gait responses of 16 healthy subjects showed more than 70% of the coefficient values are between 0.54 and 1, meaning that the majority of the healthy subjects demonstrated more than 50% of each individual intervention when combined. A coefficient of more than 1 for either $C_1$ or $C_2$ would mean adding an intervention has acted as a catalyst and emphasized the corresponding therapy method. A coefficient of zero (or close to zero) for either $C_1$ or $C_2$ would mean that the subject was not able to respond to the corresponding intervention or the effect of the corresponding intervention was lost completely or overlapped with the other intervention during the combined ARAS and SBT trials. Five subjects had a coefficient ranging between 1.06 and 1.34. This could indicate that the addition of a therapy method has made the other method more impactful. Two subjects indicated close to zero or negative coefficients during trial 1 (ARAS+tied-belt). This is not an unexpected outcome in therapies that are based on rhythmic auditory feedback. Previous research has shown that while some people have a positive response to rhythmic stimulation, others might have none or even negative response^17,37^. The performance of rhythmic stimulation has been connected to the rhythmic skills of people^37^.

If we consider a minimum of 40% as a considerable demonstration of asymmetries in gait response, more than 90% of the subjects were considerably affected from each intervention during both combined trials. While the exact coefficients for each subject might vary depending on their strengths and weaknesses, both the fitted linear model on the averaged data (Fig. 4a,b) and the close range of the whisker plots for individuals (Fig. 5) indicate that neuromusculoskeletal system can linearly combine the effects of two simultaneous rehabilitation stimuli on gait asymmetric response. Experimental results showed that the additivity principle was met; however, the coefficients not being one indicates that the homogeneity was not fully applicable. Therefore, the superposition principle was largely applied. This result can lead to a better and improved combination of therapies that accommodate the needs of patients as well as leveraging their strengths for better outcomes.

The findings from this study have the potential to significantly impact gait rehabilitation in people with neurological disorders such as stroke. Additional understanding is needed to get to this level, such as the development of accurate, reliable, and practical assessment methods to determine to what extent a patient responds to the individual or jointly applied sensory stimuli. Once the methods are ready to use, therapists can easily identify and apply optimal levels of sensory stimuli needed for personalized gait rehabilitation based on the patient’s capabilities and needs. For example, a patient, who experiences significantly altered step time but minimally impaired step length after stroke, may require a great level of auditory cue and a minimal level of visual cue to maximize gait recovery. The linear regression model introduced in this study can help develop the methods that effectively assess the patient’s responses to the sensory stimuli.

There are limitations in this study that further research can expand on. First, the interventions employed in this research (split-belt and rhythmic stimulation) engage two different mechanisms to train gait. Split-belt engages proprioceptors in lower limbs and affects interlimb control while rhythmic stimulation takes advantage of auditory sensory feedback. As a result, there is little interference in their mechanism to create an asymmetric response in gait when implementing both stimuli simultaneously. Applying interventions that engage similar sensory receptors or motor control could create non-linearity of feedback mechanism in gait. Extending the model to other therapies using tactile or somatosensory feedback as well as muscle retraining or robot-assisted training is needed to better understand the gait feedback mechanism. Another limitation of our study is that asymmetric interventions were only applied with 2:1 or 1:2 ratios (consistent with the standard of previous research with successful outcomes^8,23^). Further experiments are needed to study the effect of different asymmetric ratios. We also recruited healthy subjects since we wanted to test the hypothesis without the influence of asymmetries in an impaired gait. However, future studies will test the hypothesis among stroke survivors since the ultimate goal is to develop multiple-rehabilitation therapies that are able to retrain an impaired gait for the long-term. Moreover, incorporating three or more simultaneous interventions can extend the modeling of gait response to more than two stimuli.

This research achieved two discoveries in the path of understanding gait response. First, ARAS is able to adapt different sides of gait by applying asymmetric cues with a 2:1 ratio, meaning that auditory sensory feedback can independently access each side of lower limbs. Second, gait response for these gait parameters can be mainly modeled as a linear system with the possibility of quantifying the contribution of various stimuli at the same time.

## Supplementary Information

Supplementary Information.
