# Supplementary material for: Superposition principle applies to human walking with two simultaneous interventions
Source: Sci Rep. 2021 Apr 2;11:7465. doi: 10.1038/s41598-021-86840-9 (PMC8018974; doi:10.1038/s41598-021-86840-9)
Supplement: Supplementary file 1 — Supplementary Information. [file 41598_2021_86840_MOESM1_ESM.pdf]

Supplementary Information for

Superposition Principle Applies to Human Walking With Two Simultaneous Interventions

Fatemeh Rasouli[1]*, Seok Hun Kim[2], and Kyle B. Reed[1]

[1]Department of Mechanical Engineering, University of South Florida, Tampa, 33620, United States
[2]School of Physical Therapy and Rehabilitation Sciences, University of South Florida, Tampa, 33620, United States

*To whom correspondence may be addressed: frasouli@usf.edu

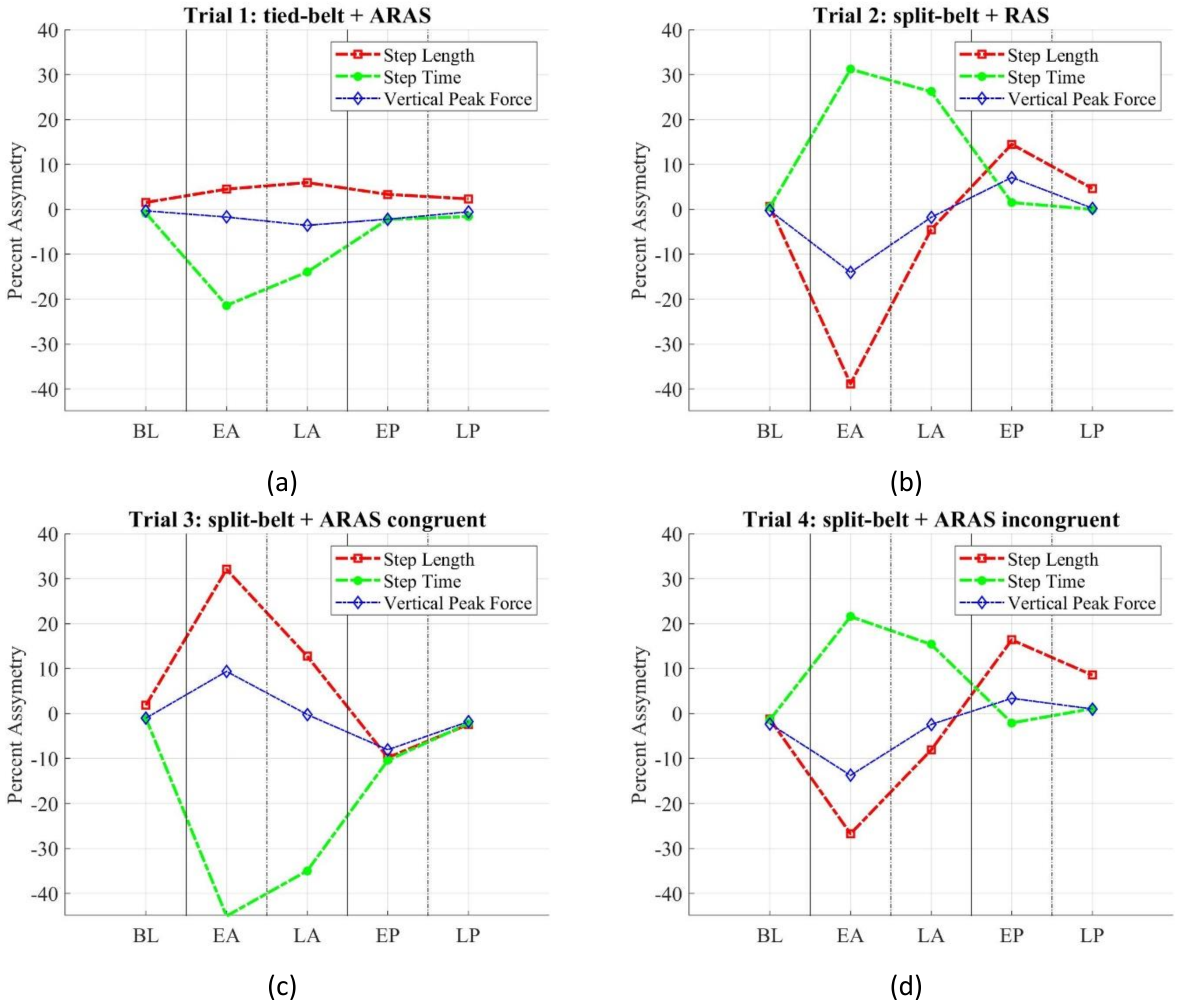

Supplementary Figure 1. Percent asymmetry average of step length, step time, and peak vertical force during five time-windows (BL: baseline, EA: early adaptation, LA: late adaptation, EP: early post-adaptation, LP: late post-adaptation) for group B. Solid vertical lines indicate the start and stop of each phase. a) trial 1: tied-belt + ARAS b) trial 2: SBT + RAS c) trial 3: SBT + ARAS congruently d) trial 4: SBT + ARAS incongruently. In trials 1 and 2 (top), only one intervention was applied asymmetrically, while both interventions were asymmetric in trials 3 and 4 (bottom).

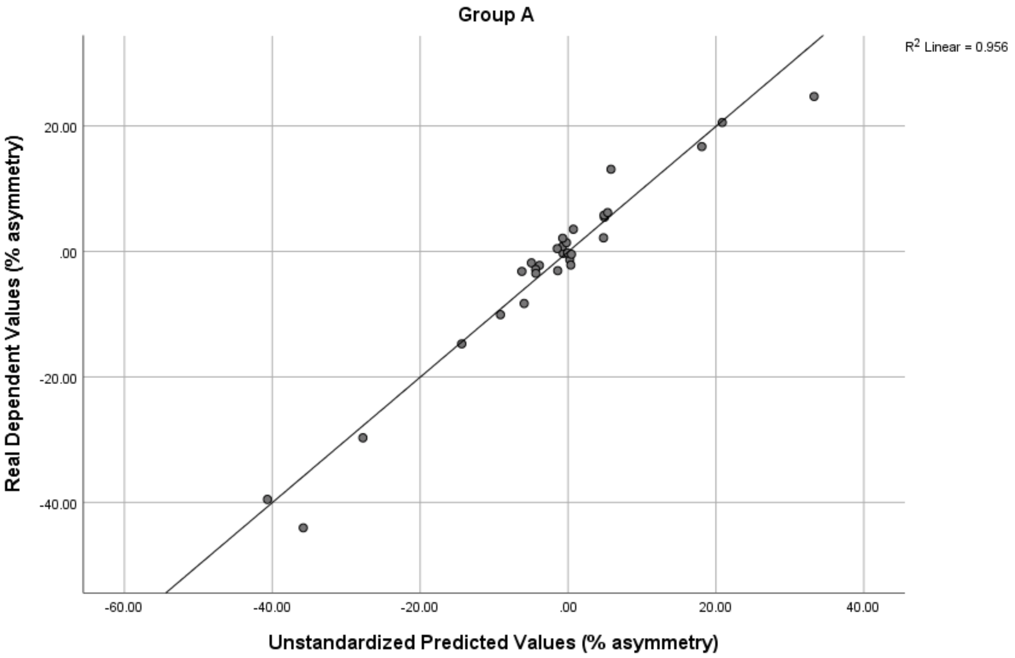

(a)

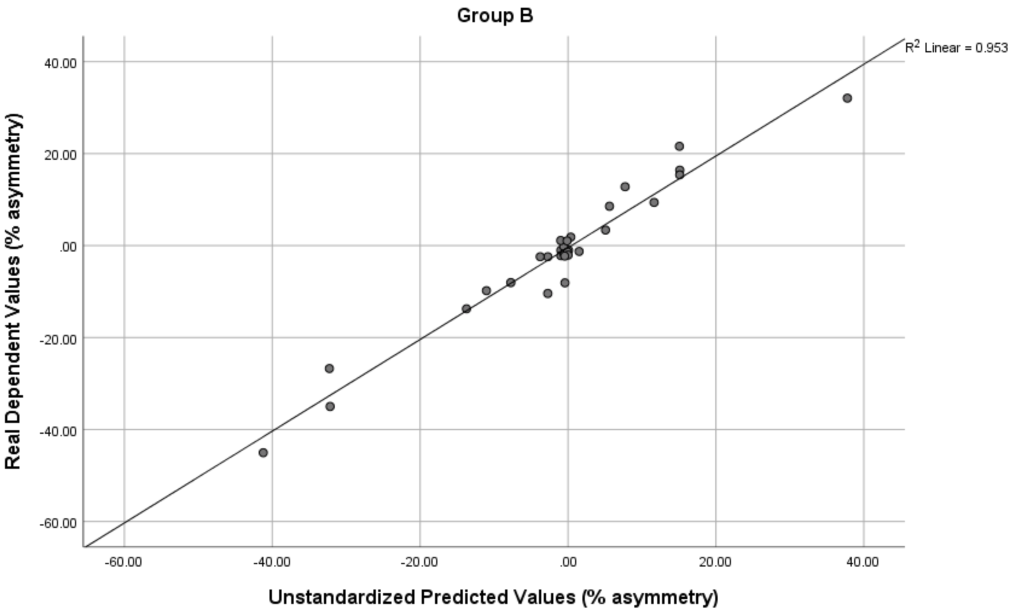

(b)

Supplementary Figure 2. Scatter plot of the predicted values from the multiple linear regression model (coefficients and independent variables) compared to the real dependent values for (a) Group A, (b) Group B. The scatter plots' density close to the linear fit shows the model accounts for substantial variance in the data.

Supplementary Table 1. Personalized coefficients for the best fit linear model of all subjects (subject ID numbers do not represent the order in which subjects were recruited. They are intentionally ordered to differentiate between the two groups).

Group A		
Sub #	C_1	C_2
1	0.48	0.57
2	0.54	1.07
3	1.14	0.75
4	0.87	0.54
5	0.64	0.7
6	0.68	0.68
7	0.6	0.75
8	0.61	1.23

Group B		
Sub #	C_1	C_2
9	0.79	1.34
10	0.65	0.84
11	0.72	0.71
12	0.96	0.76
13	0.19	0.48
14	0.88	1.06
15	-0.1	0.86
16	0.66	0.69
